# Investigation of Solubility Behavior of Canagliflozin Hydrate Crystals Combining Crystallographic and Hirshfeld Surface Calculations

**DOI:** 10.3390/molecules26020298

**Published:** 2021-01-08

**Authors:** Yefen Zhu, Yanlei Kang, Ling Zhu, Kaxi Yu, Shuai Chen, Guping Tang, Xiurong Hu

**Affiliations:** 1 Department of Chemistry, Zhejiang University, Hangzhou 310028, China; zhuyefen@hdnewdrug.com (Y.Z.); zhuling@hdnewdrug.com (L.Z.); yukaxi@zju.edu.cn (K.Y.); 3140102871@zju.edu.cn (S.C.); tangguping@zju.edu.cn (G.T.); 2 Hangzhou Huadong Medicine Group Pharmaceutical Research Institute Co., Ltd., Hangzhou 310011, China; 3 School of Information Engineering, Huzhou University, Huzhou 313000, China; 02959@zjhu.edu.cn

**Keywords:** canagliflozin, hemihydrate, monohydrate, crystal structure, solubility

## Abstract

Canagliflozin (CG) was a highly effective, selective and reversible inhibitor of sodium-dependent glucose co-transporter 2 developed for the treatment of type 2 diabetes mellitus. The crystal structure of CG monohydrate (CG-H_2_O) was reported for the first time while CG hemihydrate (CG-Hemi) had been reported in our previous research. Solubility and dissolution rate results showed that the solubility of CG-Hemi was 1.4 times higher than that of CG-H_2_O in water and hydrochloric acid solution, and the dissolution rates of CG-Hemi were more than 3 folds than CG-H_2_O in both solutions. Hirshfeld surface analysis showed that CG-H_2_O had stronger intermolecular forces than CG-Hemi, and water molecules in CG-H_2_O participated three hydrogen bonds, forming hydrogen bond networks. These crystal structure features might make it more difficult for solvent molecules to dissolve CG-H_2_O than CG-Hemi. All these analyses might explain why the dissolution performance of CG-Hemi was better than CG-H_2_O. This work provided an approach to predict the dissolution performance of the drug based on its crystal structure.

## 1. Introduction

Polymorphs in organic crystals involve the formation of isomeric molecular identity. The polymorph phenomenon refers to the existence of more than two kinds of crystalline states for a compound and the state is also called “isomorphism”. Polymorphs are widely used in medicine, dyes, food additives, agricultural chemicals and other fields. From a scientific and commercial point of view, the issue of polymorphism remains important. Different crystal forms appear in the process of drug production. In the process of drug production, polymorphism is of great importance as different crystal forms of the drug can show different stability, solubility, dissolution rate and bioavailability, especially for poorly soluble drugs [1]. The solubility and dissolution rate of an oral drug will help decide whether it will generate high systemic bioavailability. In the industrial development of a new drug, the candidate drug with poor water solubility will cause considerable problems because of its low bioavailability. Therefore, systematic screening of polymorphs has become an essential step in drug development [2].

Canagliflozin (CG) is a selective and reversible inhibitor of sodium-glucose cotransporter 2 (SGLT2) for the treatment of type 2 diabetes mellitus (T2DM), which can reduce glycemia as well as blood pressure, body weight and albuminuria in people with diabetes [3,4]. CG is chemically defined as (1*S*)-1,5-anhydro-1-[3-[[5-(4-fluorophenyl)-2-thienyl]methyl]-4-methylphenyl]-d-glucitol. Based on the low solubility and low permeability, CG is classified as class IV according to Biopharmaceutics Classification System (BCS) [5,6]. CG is mainly marketed in the form of solid dosage forms, especially tablets. So, it’s necessary to study the solubility and dissolution properties of CG. There are several crystalline forms of CG that have been reported in the patent, such as CG form A [7], CG form B [8], CG form C [9], CG form D [9], CG form E [10], CG form F [10], CG monohydrate [10] and CG hemihydrate [11]. Among them, crystalline form of CG-hemihydrate and monohydrate can be found as the more frequently polymorphs. Generally, the most thermodynamically stable form is usually chosen for pharmaceutical use. It was reported that in order to overcome dissolution and bioavailability constrains, the currently marketed formulation contained CG as a hemihydrate form [12].

However, it has been found that the dissolution rate of CG hemihydrate (CG-Hemi) deteriorates during the preparation and storage process (especially under high humidity conditions). The reason is that the CG-Hemi is partially or completely transformed into CG monohydrate (CG-H_2_O) during storage or preparation. So the purpose of this article is to find out the reasons for the difference in solubility and dissolution rate between CG-Hemi and CG-H_2_O by studying on the crystal structures of these two crystal forms, and to find a way to make CG-Hemi more stable.

## 2. Results

### 2.1. Crystal Structure of CG-Hemi and CG-H_2_O

**Figure 1:**
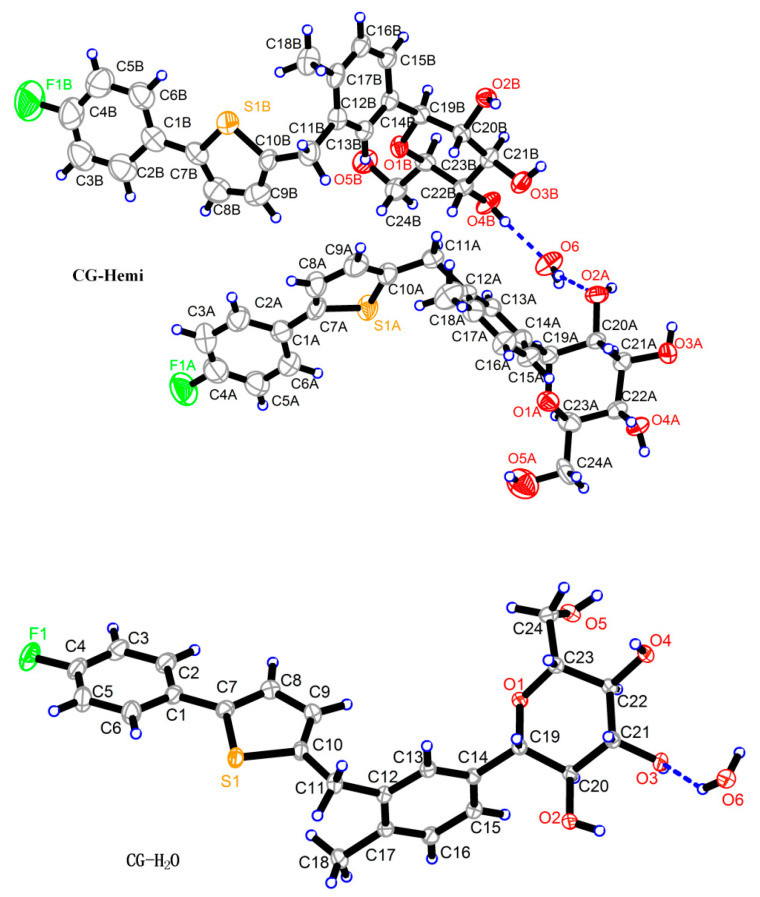
ORTEP (Oak Ridge Thermal Ellipsoid Plot Program) diagrams of CG-Hemi and CG-H_2_O. There are two CG molecules and one water molecule in CG-Hemi. There are one CG molecule and one water molecule in CG-H_2_O.

**Figure 2:**
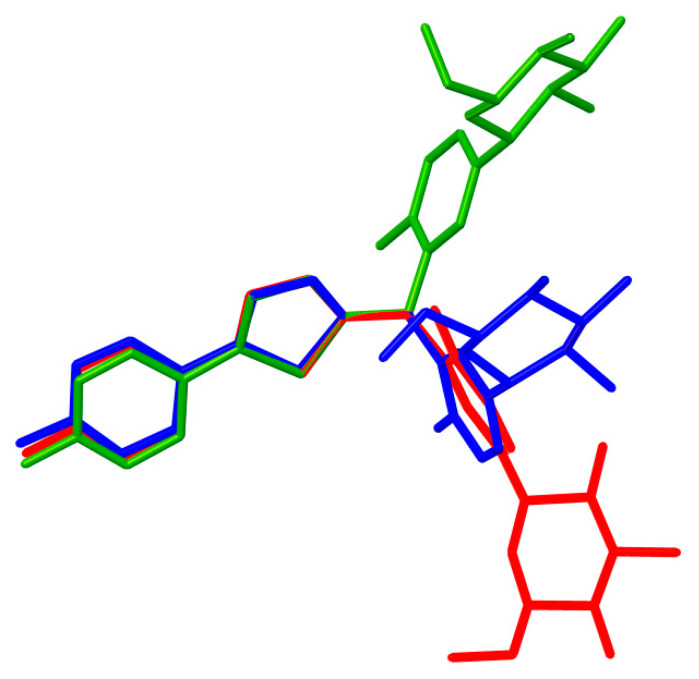
Molecules overlay of CG-Hemi (blue and red) and CG-H_2_O (green). H atoms have been omitted for clarity.

The crystal structure of CG-Hemi had been reported before [13], and the crystal structure of CG-H_2_O was reported for the first time. The crystal structures of CG-Hemi and CG-H_2_O were determined by single crystal X-ray analysis and the ORTEP (Oak Ridge Thermal Ellipsoid Plot Program) diagram of CG-Hemi and CG-H_2_O were shown in Figure 1. CG-Hemi crystallized in the P2_1_2_1_2_1_ space group, with the asymmetric unit consisting of two CG molecules and one water molecule. In the asymmetric unit of CG-H_2_O, there were one CG molecule and one water molecule, with space group of P2_1_. Crystal data, collection and structure refinement details of CG-Hemi and CG-H_2_O were summarized in Table 1. CG molecule was flexible in the crystal structure. Two CG molecules in CG-Hemi and one CG molecule in CG-H_2_O were overlaid in Figure 2. It could be seen that benzene ring and thiophene ring in the three molecules were almost overlapping. The main differences of them were the configuration of methyl]-4-methylphenyl]-d-glucitol, which are shown from the torsion angles of the three molecules and the directions of the three benzene rings connected to the C11 atom.

**Figure 3:**
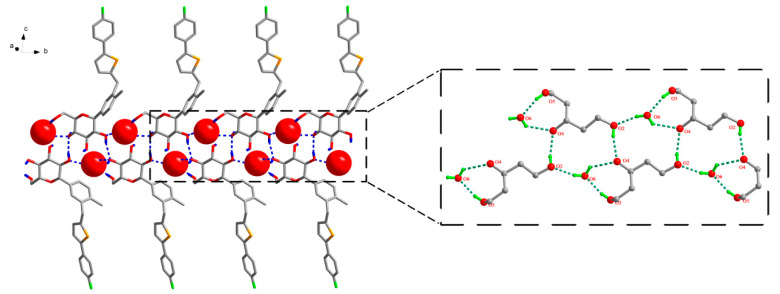
Water molecule and CG molecule interlinked by hydrogen bonds in the CG-H_2_O crystal structure. There were two types of ring motifs, i.e., $R_{4}^{4}$(15), $R_{2}^{4}$(7) along b axis. Hydrogen bonds were represented by dashed lines. For clarity, hydrogen atoms not involving in hydrogen bonds were omitted.

The analysis of the strong hydrogen bonding pattern showed that in the crystal structure of CG-H_2_O, two adjacent CG molecules formed CG dimers by intermolecular hydrogen bond O2–H2···O4 (Symmetry code: −1 − x, 1/2 + y, Figure 3). Then these dimers were linked with abundant hydrogen bonds between CG molecules and water molecules along b-axis: O3–H3···O6 (Symmetry code: 1 + x, 1 + y, 1 + z), O5–H5···O6 (Symmetry code: −1 − x, −1/2 + y, −z), O6–H6A···O3 and O6–H6B···O2 (Symmetry code: −1 − x, −1/2 + y, −z). All H atoms of OH group and water molecules participated in the strong intermolecular O–H···O hydrogen bonds and generated supramolecular self-assembly $R_{4}^{4}$(15), $R_{2}^{4}$(7) ring motifs along b axis in CG-H_2_O and formed a two-dimensional structure (Figure 3), which were further connected by the hydrogen bond O4–H4···O5 (Symmetry code: −1 + x, 1 + y, 1 + z). Details of the hydrogen bond geometry of CG-H_2_O were listed in Table 2.

**Figure 4:**
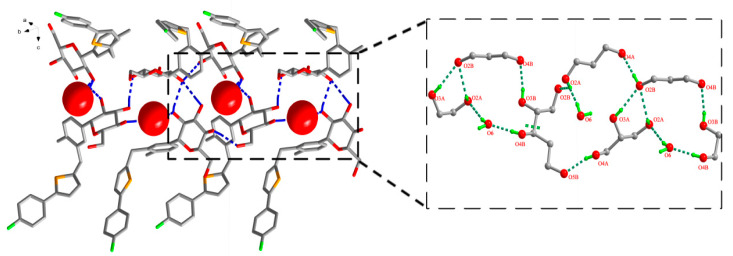
Water molecule and CG molecule interlinked by hydrogen bonds in the CG-Hemi crystal structure. There were three types of ring motifs along b axis, $R_{4}^{4}$(15), $R_{2}^{1}$(7) and $R_{4}^{4}$(21). Hydrogen bonds were represented by dashed lines. For clarity, hydrogen atoms not involving in hydrogen bonds were omitted.

In the case of CG-Hemi, hydrogen bonds between water molecules and CG molecules formed three types of ring motifs $R_{4}^{4}$(15), $R_{2}^{1}$(7) and $R_{4}^{4}$(21) along b axis (Figure 4). Obviously, water molecules in CG-Hemi participated in the formation of two hydrogen bonds, while in CG-H_2_O, water molecules participated in the forming of three hydrogen bonds (Figure 3 and Figure 4).

### 2.2. Solubility and Dissolution Study

**Figure 5:**
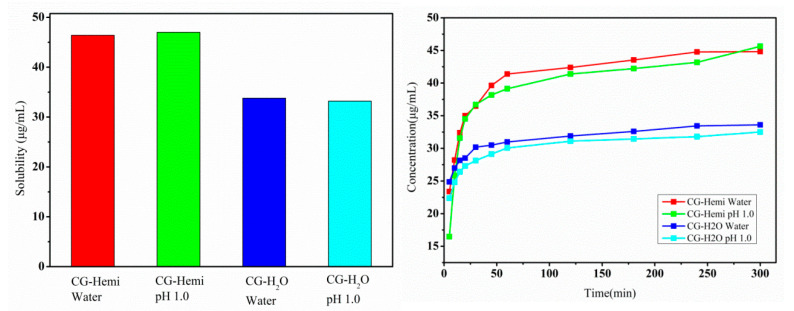
Solubility (**left**) and dissolution study (**right**) results of CG-Hemi and CG-H_2_O.

Equilibrium solubility and dissolution rate of drugs were two important parameters in pharmaceutical development and quality control. The equilibrium solubility and dissolution rate of CG-Hemi and CG-H_2_O were studied both in water and HCl solution (pH 1.0). As shown in Figure 5, the equilibrium solubility of CG-H_2_O was 33.9 μg/mL in water and 33.2 μg/mL in HCl solution (pH 1.0). Compared to CG-H_2_O, CG-Hemi obtained a higher solubility (46.4 μg/mL in water and 47.0 μg/mL in HCl solution). Therefore, the solubility of CG-Hemi was 1.4 times higher than that of CG-H_2_O in both solutions. The dissolution data within 20 min for CG-Hemi and CG-H_2_O were used to calculate the slope of the curve, which represented the intrinsic dissolution rate. In our experiment, intrinsic dissolution rate of CG-Hemi (0.78 μg·mL^−1^·min^−1^) in water was 3.25 folds faster than that of CG-H_2_O (0.24 μg·mL^−1^·min^−1^), and the intrinsic dissolution rate of CG-Hemi (1.20 μg·mL^−1^·min^−1^) was 3.6 times of CG-H_2_O (0.33 μg·mL^−1^·min^−1^) in HCl solution. The dissolution study results showed that CG-Hemi dissolved more quickly than CG-H_2_O both in two solutions.

### 2.3. Hirshfeld Surface Analysis

**Figure 6:**
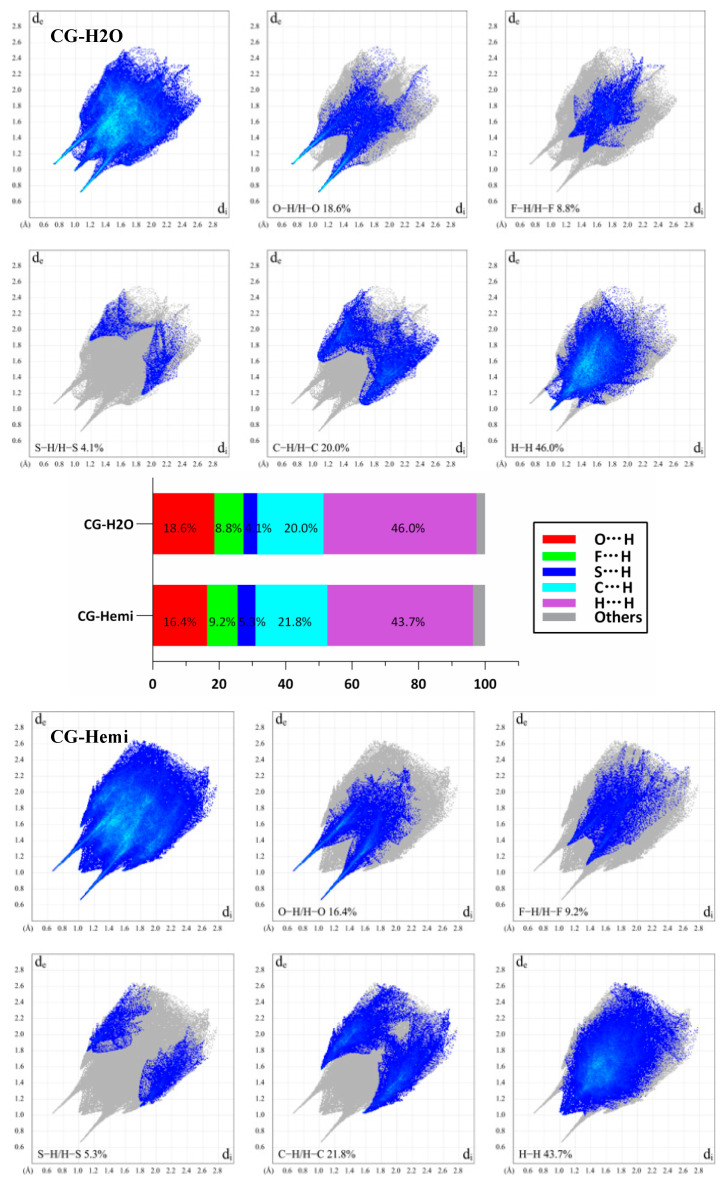
2D fingerprint plots of the Hirshfeld surfaces for CG-H_2_O (upper) and CG-Hemi (lower). The relative percentage contributions to the Hirshfeld surface area of various intermolecular contacts for CG-H_2_O and CG-Hemi (middle). d_e_ is the distance from the point to the nearest nucleus external to the surface, and d_i_ is the distance to the nearest nucleus internal to the surface.

The two-dimensional fingerprint plots of Hirshfeld surface analysis were used to compare the intermolecular interactions between CG-H_2_O and CG-Hemi. The software of Crystal Explorer 17.5 was used for the calculations [14,15]. As shown in Figure 6, the results showed that H···H interactions were dominant both in the crystal structure of CG-H_2_O and CG-Hemi. The percentage of O···H/H···O interactions and H···H interactions for CG-H_2_O were higher than that of CG-Hemi, and F···H/H···F, S···H/H···S and C···H/H···C interactions for CG-H_2_O were lower than that of CG-Hemi. The intermolecular forces included hydrogen bonds, van der Waals forces, π···π interactions, etc. Among them, O···H/H···O interactions for CG-H_2_O mainly came from hydrogen bonding (O2–H2···O4, symmetry code: −1 − x, 1/2 + y; O3–H3···O6, symmetry code: 1 + x, 1 + y, 1 + z; O5–H5···O6 symmetry code: −1 − x, −1/2 + y, −z; O6–H6A···O3 and O6–H6B···O2, symmetry code: −1 − x, −1/2 + y, −z; O4–H4···O5, symmetry code: −1 + x, 1 + y, 1 + z). However, H···H interactions for CG-H_2_O might come from hydrogen bonding and van der Waals force, because there was no strong π···π stacking interaction in the crystal structure of CG-H_2_O. Therefore, we could include that CG-H_2_O had stronger intermolecular interactions (including hydrogen bonding and van der Waals force) than CG-Hemi [16].

**Figure 7:**
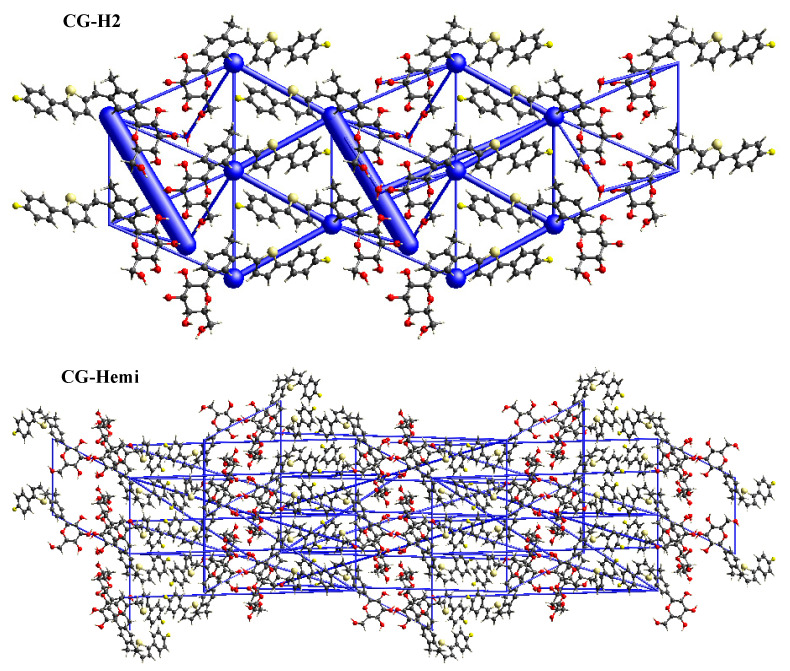
Total energy frameworks diagram of CG-H_2_O and CG-Hemi.

In order to study, the packing of the crystal structure and the supramolecular rearrangement of CG-H_2_O and CG-Hemi, total energy framework diagrams of CG-H_2_O and CG-Hemi were shown in Figure 7 along a-axis. Total energy included four components of electrostatic (E_ele_), polarization (E_pol_), dispersion (E_dis_), and repulsion (E_rep_), and was calculated at the B3LYP/6-31G (d,p) level using 3.8 Å radius cluster of molecules. Blue cylinders represented the cylinder radius which was proportional to the strength of the interaction energy. The two longest and thickest blue cylinders in the energy frameworks of CG-H_2_O represented the hydrogen bonds between the water molecules and CG molecules. Other thicker blue cylinders represented the van der Waals force in the crystal structure of CG-H_2_O. The energy frameworks of CG-Hemi were thinner and more average than CG-H_2_O, which meant lower strength of the interaction energy of CG-Hemi.

### 2.4. PXRD

**Figure 8:**
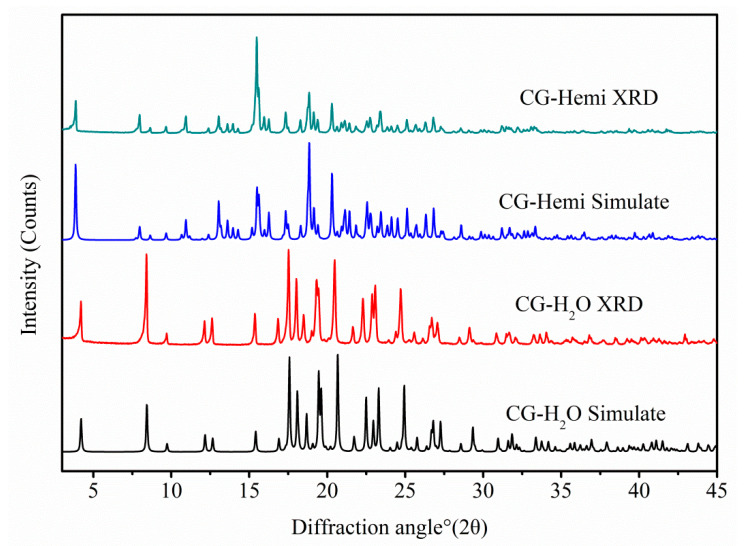
Experimental PXRD patterns and simulated PXRD patterns from the crystal structure of CG-Hemi and CG-H_2_O.

Overlay of experimental and calculated PXRD patterns of CG-Hemi and CG-H_2_O were shown in Figure 8, and each form had a distinguishable PXRD pattern. The characteristic peaks of CG-Hemi were 3.87, 7.96, 8.64, 9.66, 10.94, 15.48, 17.34, 18.74, 19.14, 20.30 diffraction angle (2θ). CG-H_2_O had characteristic peaks of 2θ (°) 4.20, 8.40, 9.70, 12.62, 15.36, 16.84, 19.32, 23.08. The experimental PXRD patterns of both forms were in good agreement with the simulated XRD patterns, which further verified the purity of the samples of CG-Hemi and CG-H_2_O.

## 3. Discussion

**Figure 9:**
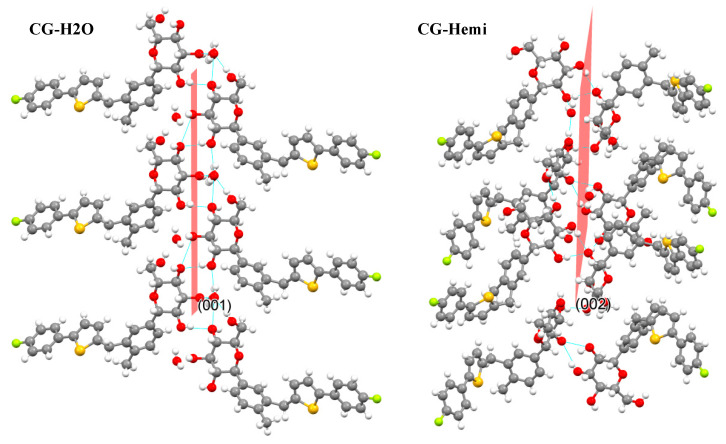
Crystal packing of CG-H_2_O along (001) face and crystal packing of CG-Hemi along (002) face.

**Figure 10:**
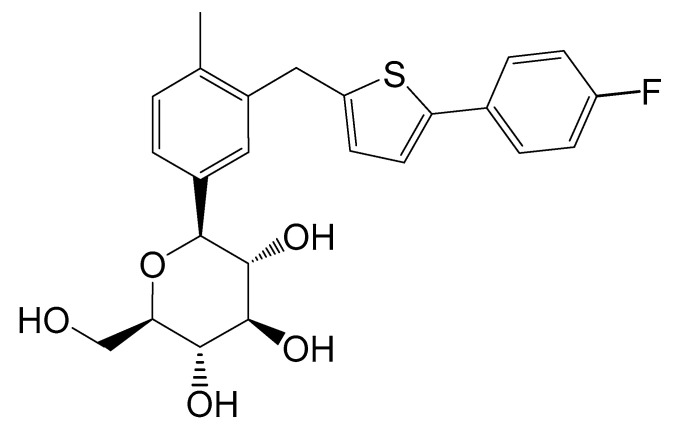
Chemical structure of CG. The chemical name of CG was (1*S*)-1,5-anhydro-1-[3-[[5-(4-fluorophenyl)-2-thienyl]methyl]-4-methylphenyl]-d-glucitol.

The polymorph CG-H_2_O had been synthesized and the crystal structure was studied by using single X-ray diffraction. PXRD results of CG-H_2_O and CG-Hemi was consistent with the simulated diffraction pattern from single X-ray diffraction, which proved the purity of the drug used in this study. The in vitro experiments showed that the solubility of CG-Hemi was 1.4 times higher than that of CG-H_2_O both in water and HCl solution. And the dissolution rates of CG-Hemi were more than 3 folds than CG-H_2_O in both solutions. The percentage of O···H/H···O interaction for CG-H_2_O was higher than that of CG-Hemi, which might relate to stronger intermolecular forces for this compound. These strong intermolecular forces made it difficult to dissociate and interact with solvent molecules, resulting in poor solubility and slow dissolution rate. The results of energy frameworks analysis also showed that CG-H_2_O had stronger intermolecular forces including hydrogen bonds and van der Walls forces than CG-Hemi. In addition, unlike in CG-Hemi, water molecules participated in the formation of two hydrogen bonds, and water molecules participated in forming three hydrogen bonds in CG-H_2_O. The water molecules in CG-H_2_O received more intermolecular forces than in CG-Hemi. During the dissolution process of CG-Hemi and CG-H_2_O, the solute molecules need to compete with water molecules in crystal structure to break the existing hydrogen bonds and form new hydrogen bonds between solution molecules and CG molecules. So, it was more difficult for solute molecules to dissolve CG-H_2_O than CG-Hemi. The dominant (001) crystal face of CG-H_2_O had a layer of water molecules and hydroxyl groups (Figure 9), and all positions that could form hydrogen bonds in this layer were occupied by hydrogen bonds. On the other hand, the dominant (002) crystal face of CG-Hemi also included water molecules and hydroxyl groups (Figure 10), but there were still some positions that could form hydrogen bonds which were not occupied. So, it could be predicted that CG-Hemi had a better solubility than CG-H_2_O. All these crystal structure characteristics explained why CG-H_2_O exhibited lower solubility than CG-Hemi.

## 4. Materials and Methods

### 4.1. Materials

CG-Hemi was provided by Huadong Pharmaceutical Co., Ltd. (Zhejiang, China) and used without further purification. CG-H_2_O was crystallized from mixture solvent of methanol and water by cooling crystallization method. The single crystals of CG-Hemi and CG-H_2_O were prepared by solvent slow evaporation method at ambient humidity and temperature. The chemical structure of CG was shown in Figure 10.

### 4.2. Single Crystal X-ray Structural Analysis

The crystal structures of CG-Hemi and CG-H_2_O were determined by using Rigaku R-AXIS-RAPID X-ray single crystal diffractometer (Rigaku, Tokyo, Japan) equipped with an imaging plate area detector and graphite monochromatic Mo-Kα radiation (λ = 0.71069 Å). Data reduction was performed with Crystal Structure [17]. The crystal structure was solved with direct methods using SHELX-S97 program (Sheldrick, G.M. SHELXS-97, Program for the Solution of Crystal Structures, University of Göttingen, Göttingen, Germany, 1997) and refined anisotropically (non-hydrogen atoms) by full-matrix least-squares method on F2 using the SHELX-L97 program (Sheldrick, G.M. SHELXS-97, Program for the Solution of Crystal Structures, University of Göttingen, Göttingen, Germany, 1997) [17]. Hydrogen atoms were found in difference Fourier-map, but placed at calculated positions and refined using the riding model. The crystal structure diagrams of CG-Hemi and CG-H_2_O were drawn by ORTEP (Visualization of crystal structure in Oak Ridge National Laboratory, 2015) [18] and Diamond (Crystal impact GBR, University of Bonn, Germany, 2005) [19,20].

### 4.3. Hirshfeld Surface Analysis

Crystallographic information file (CIF) of CG-Hemi and CG-H_2_O were used for the Hirshfeld surface analysis using the program CrystalExplorer17.5 [14]. Information on the relative contribution to the Hirshfeld surface could be plotted in a two-dimensional graphical view of points with de and di distance scales. This is a so-called two-dimensional fingerprints, which simultaneously analyzed all molecular interactions. In order to generate a fingerprint, the bond length of the hydrogen atoms participating in the interaction was standardized to the standard neutron value (C–H = 1.083 Å, O–H = 0.983 Å). Then energy framework analysis was used to explore the intermolecular interaction energies between the molecules of the cluster within 3.8 Å at the theoretical level of B3LYP/6-31G (d,p).

### 4.4. Powder X-ray Diffraction

The Powder X-ray diffraction (PXRD) results were obtained on a Rigaku D/Max-2550 powder diffractometer (Rigaku Co., Tokyo, Japan), with a CuKα radiation source, λ = 1.54059 Å and operated at 40 kV and 250 mA. The scans were ran from 3.0 to 50.0° (2θ), with an increasing step size of 0.02° (2θ) and count time of 2 s per step. Data were processed using the MDI-Jade version 9.0 software (Philadelphia, PA, USA).

### 4.5. Solubility Measurements

The solubility and dissolution rate of CG-Hemi and CG-H_2_O was measured by SHIMADZU UV-2450 spectrometer (Kyoto, Japan). The obtained powders of CG-Hemi and CG-H_2_O were passed through a 100-mesh sieve. In this study, solubility was evaluated in two media: hydrochloric acid solution (HCl solution, pH 1.0) to represent stomach conditions and water. Excess quantities of samples (0.2 g) were dispersed in 10 mL of water or HCl solution (pH 1.0) at 150 rpm, 37 °C for 24 h to obtain saturated solutions. For dissolution rate determination, excess quantities of drugs were added into 900 mL water or HCl solution (pH 1.0) and rotated at 150 rpm at 37 °C. Samples (10 mL) were collected at 5, 10, 15, 20, 30, 45, 60, 120, 180, 240, 300 min using an automatic sampler and replaced with an equal volume of the medium solution to maintain a constant total volume. The final powders in the dissolution study were determined by PXRD and compared with CG-Hemi or CG-H_2_O. All solutions were filtered with 0.45 μm Millipore Millex-HV Hydrophilic PVDF filter (Danvers, MA, USA) and measured at their λ_max_.

## 5. Conclusions

In summary, CG-H_2_O and CG-Hemi were synthesized and the chemical identities of two crystal forms were confirmed by PXRD. The stability of CG-H_2_O is better than that of CG-Hemi under high humidity conditions, because CG-Hemi would be converted into CG-H_2_O under this condition. Solubility and dissolution rate results showed that the equilibrium solubility of CG-Hemi was 1.4 times higher than that of CG-H_2_O in water and HCl solution, and the dissolution rates of CG-Hemi were more than 3 folds higher than CG-H_2_O in both solutions. Crystal structure and Hirshfeld surface analysis displayed that CG molecules and water molecules in CG-H_2_O formed stronger intermolecular forces than CG-Hemi. In the two-dimensional fingerprint plot results, a higher ratio of O···H/H···O interactions and H···H interactions for CG-H_2_O might indicate stronger intermolecular interactions (including hydrogen bonding and van der Waals force) than CG-Hemi. Energy frameworks results also showed stronger intermolecular interactions of CG-H_2_O than that of CG-Hemi. These results all explained the lower solubility and dissolution rate of CG-H_2_O than CG-Hemi. It showed that the dissolution performance of the drug crystal form could be inferred by the crystal structure.

## Figures and Tables

**Table 1:** Crystal parameter data of CG-Hemi and CG-H_2_O.

Crystal Data	CG-Hemi (Reported by Liu [13])	CG-H_2_O
CCDC number	1475516	2022368
Chemical formula	2(C_24_H_25_FO_5_S)·H_2_O	C_24_H_25_FO_5_S·H_2_O
Molecular weight	907.02	462.51
Crystal system	Orthorhombic	Monoclinic
Space group	P2_1_2_1_2_1_	P2_1_
Temperature (K)	296	296
a, b, c (Å)	8.4259(4),11.4264(7), 45.706(2)	5.1280(3), 10.0824(5), 21.0305(12)
α, β, γ (°)	90, 90, 90	90, 94.586(2), 90
V(Å^3^)	4400.4(4)	1083.85(10)
Z	4	2
Radiation type	Mo Kα	Mo Kα
Μ (mm^−1^)	0.192	0.198
Crystal size (mm)	0.48 × 0.28 × 0.26	0.39 × 0.23 × 0.18
**Data collection**		
Diffractometer		
Diffraction wavelength	0.71073 Å	0.71073 Å
Absorption correction	Multi-scan	Multi-scan
T_min_,T_max_	0.9136, 0.9518	0.7068, 0.7455
No. of measured, independent and observed [I > 2σ(I)] reflections	43211, 9958, 5079	25194, 4762, 4691
R_int_	0.1447	0.0239
(sinθ/λ)_max_(Å^−1^)	0.1274	0.0196
**Refinement**		
R [F^2^ > 2α(F^2^)], ωR(F^2^), S	0.0800, 0.1166, 0.999	0.0245, 0.0653, 1.066
No. of reflections	9958	4762
No. of parameters	575	292
H-atom treatment	constraint	constraint
Δρ_max_, Δρ_min_ (e Å^−3^)	0.38, −0.29	0.28, −0.18

**Table 2:** Intermolecular hydrogen bond geometry for CG-H_2_O.

D–H···A	D–H (Å)	H···A (Å)	D···A (Å)	Angle (°)
O3–H3···O6^i^	0.84	1.95	2.7890(18)	178.9
O2–H2···O4^ii^	0.84	2.09	2.9172(17)	166.9
O5–H5···O6^iii^	0.84	2.00	2.8087(19)	161.9
O4–H4···O5^iv^	0.84	1.95	2.7648(19)	163.0
O6–H6A···O3	0.87	2.11	2.8271(18)	139.0
O6–H6B···O2^iii^	0.87	2.00	2.8425(19)	162.4

Symmetry transformations used to generate equivalent atoms: (i) 1 + x, 1 + y, 1 + z; (ii) −1 − x, 1/2 + y, −z; (iii) −1 − x, −1/2 + y, −z; (iv) −1 + x, 1 + y, 1 + z.

## Data Availability

The data presented in this study are available on request from the corresponding author. The data are not publicly available due to privacy.
